# Can Interpersonal Behavior Influence the Persistence and Adherence to Physical Exercise Practice in Adults? A Systematic Review

**DOI:** 10.3389/fpsyg.2018.02141

**Published:** 2018-11-06

**Authors:** Filipe Rodrigues, Teresa Bento, Luís Cid, Henrique Pereira Neiva, Diogo Teixeira, João Moutão, Daniel Almeida Marinho, Diogo Monteiro

**Affiliations:** ^1^Department of Sports Sciences, Beira-Interior University, Covilhã, Portugal; ^2^Research Center in Sport Sciences, Health Sciences and Human Development, Vila Real, Portugal; ^3^Sport Science School of Rio Maior (ESDRM-IPSantarém), Rio Maior, Portugal; ^4^Faculty of Physical Education and Sport (ULHT), Lusófona University, Lisbon, Portugal

**Keywords:** self-determination theory, physical exercise, interpersonal behaviors, motivation, persistence, adherence

## Abstract

**Objective:** Motivation seems to be a fundamental indicator of long-term physical exercise adherence. Self-Determination Theory (SDT) argues that social environment plays a central role in the satisfaction of basic psychological needs, which might directly affect the quality of one's motivation. Individuals who appear to be more self-determined tend to persist longer at certain behaviors. Therefore, this body of work intends to analyze the relationship between motivational variables and behavioral outcomes in the exercise context, having as theoretical background the Self-Determination Theory.

**Methods:** This systematic review was conducted through an electronic search on Web of Science, PubMed, SPORTDiscus, and PsycINFO. Data such as instruments, main predictors and results were collected from studies published between 1985 and 2018. A total of 35 empirical studies were considered for a detailed analysis.

**Results:** Results showed the relevance of autonomy support performed by exercise professionals, as well as the major contribution that these behaviors have toward the satisfaction of basic psychological needs, besides the inherent benefits of developing more autonomous regulations. According to the literature, few studies have analyzed interpersonal thwarting behavior and the way this relates to basic psychological needs' frustration. Nether less, there seems to be a negative relationship between less self-determined regulations and exercise practice.

**Conclusion:** Despite the existence of numerous cross-sectional studies that demonstrate positive correlations between SDT and behavioral outcomes in the exercise context, longitudinal research that analyzes all six dimensions of interpersonal behaviors and their relationship with persistence and adherence to exercise proves to be crucial. However, according to this review, interventions based on SDT appear to be fundamental when it comes to promote the maintenance of a long-term exercise practice.

## Introduction

Physical inactivity is currently one of the largest changeable risk behaviors, being the fourth largest risk factor contributing to death (World Health Organization, 2017). As a matter of fact, approximately 3.2 million people die each year from chronic diseases associated with these behaviors. According to Eurobarometer (2018), the main reasons pointed out by people to justify physical inactivity were “lack of time” and “lack of motivation,” respectively by 43 and 23%. Caudwell and Keatley (2016) argue that both motives are associated to the psychological state of amotivation, meaning that the person does not feel motivated or lacks of intention to exercise. These high percentages of physical inactivity may be linked to health professionals' (i.e., exercise professionals) behaviors, who use overly forced and commercial approaches, perceiving people only as clients and ignoring their human component (Teixeira et al., 2012; Santos et al., 2016). The social environment works as a source of personal fulfillment, ultimately contributing to enhance one's motivation quality and consequently, playing a fundamental role in the maintenance of physical activity practice (Hagger and Chatzisarantis, 2008).

Among several theories that analyze motivation, Self-Determination Theory (SDT) stands out by focusing on the personality factors, the surrounding context, as well as on the causes and consequences of self-determined behavior (Deci and Ryan, 2000). This conceptual framework has been applied in several contexts, namely in education (Grangeia et al., 2016), physical education (Standage et al., 2005), sports (Rocchi and Pelletier, 2018), and also in the exercise context (Teixeira et al., 2012; Phillips and Johnson, 2017). In addition, some studies (Ntoumanis, 2001; Teixeira et al., 2018) claim that SDT is the most widely motivational construct used by researchers on understanding the influence of human motivation on behavior outcomes in the exercise context.

SDT postulates the existence of three basic psychological needs (BPN; autonomy, competence, and relatedness) innate in all human beings, whose satisfaction translates into a universal experience of physical and psychological well-being (Ryan and Deci, 2000b). The BPN's satisfaction is a strong predictor of more self-determined motivation (Edmunds et al., 2007; Vallerand and Young, 2014; Chen et al., 2015), therefore being associated with several positive outcomes at behavioral, cognitive, and affective level (Deci and Ryan, 2000; Wilson et al., 2002; Edmunds et al., 2006). Contrarily, BPN's frustration is tied up, in various contexts, with less self-determined forms of motivation, which might lead to inhibition of personal and human development (Bartholomew et al., 2011b). It is worth to mention that BPN satisfaction and frustration should be seen as independent constructs, and not as cause-effect between low levels of satisfaction and high levels of frustration (Bartholomew et al., 2011a). When it comes to the exercise context, BPN satisfaction turns out to be a strong predictor of intrinsic motivation (Ryan and Deci, 2000a), being ultimately related with long-term exercise adherence (Teixeira et al., 2012). On the contrary, BPN's frustration predicts amotivation (Bartholomew et al., 2011a; Vansteenkiste and Ryan, 2013), leading to low adherence and high dropout rates (Bartholomew et al., 2011b; Ng et al., 2013).

Ryan and Deci (2017) state that the level of motivation depends on the satisfaction of BPN's and that, instead of a dichotomous (intrinsic vs. extrinsic) response, motivation can be manifested in six different ways. The different motivational regulations are spread along a motivational continuum, ranging from amotivation (i.e., lack of motivation or lack of intention to act accordingly to a given behavior) to intrinsic motivation (i.e., pleasure underlying a particular behavior), the last one representing the prototype of self-determined behavior. Extrinsic motivation arises in the middle of this continuum and includes four different types of regulation, two of which are more self-determined (i.e., autonomous regulation): identified regulation (i.e., the individual recognizes the importance of the activity, although he/she may not enjoy to perform it) and integrated regulation (i.e., the person integrates the behavior as inherent to him/herself and perceives it as being aligned with his own values); and other two less self-determined (i.e., controlled regulation): external regulation (i.e., the person performs the behavior in order to satisfy external requirements) and introjected regulation (i.e., the person pressures him/herself to perform the behavior).

This distinction between autonomous and controlled regulation is the core characteristic of the SDT (Ryan and Connell, 1989). Moreover, this theory describes the process responsible for the shift from controlled regulations toward more internalized behaviors, as well as the impact that different regulations have on the behavior itself (Deci and Ryan, 2008; Howard et al., 2017). Previous research in the exercise context emphasizes the relationship between the degree of autonomous regulation and several positive behavioral outcomes, such as increased enjoyment (Ruby et al., 2011), and higher levels of persistence and adherence (Fortier et al., 2007; Vlachopoulos and Neikou, 2007). On the contrary, less self-determined regulations require external motivational sources to perform a specific behavior, making behavior withdrawal more likely to occur (Ryan and Deci, 2000a).

Enjoyment has been described as the process of experiencing satisfaction, joy and pleasure during the performance of a particular behavior (Dacey et al., 2008). Thus, it is also considered to be significant predictor of exercise practice (Moreno-Murcia et al., 2012; Vallerand and Young, 2014). Else ways, controlled regulations tend to lead to the opposite emotional state, characterized by boredom, disinterest and dropout (Teixeira et al., 2018).

Research on the intention to exercise has shown that the exerciser's behavior only becomes a habit when it is maintained for at least 6 months after the intervention has started (Pavey et al., 2011). Chatzisarantis and Hagger (2009) developed an intervention aiming to analyze the changes in exercise adherence in students during leisure time. Results showed that the intervention group had a greater intention to maintain exercise practice in their free time and affirmed to practice more hours of physical activity, compared to the control group. In another study (Silva et al., 2010) based on SDT, conducted in order to analyze exercise frequency, researchers observed that the target group of intervention demonstrated higher levels of persistence and lower fat mass percentage. These findings suggest that SDT framework can be effective in the internalization of exercise practice, facilitating the persistence and adherence to the behavior.

As previously mentioned, SDT refers to the social context as a predictor of human behavior, since individuals are constantly extracting information from the surrounding people, in order to interpret their own behaviors. Beyond doubt, interpersonal behaviors play a major role in the satisfaction and/or frustration of BPN's, allowing to predict how motivation is regulated (e.g., Bartholomew et al., 2011b). Therefore, social interactions highly impact human motivation in several life aspects (Deci and Ryan, 1985).

According to SDT, people may perceive six different interpersonal behaviors (Rocchi et al., 2017): autonomy support (i.e., freedom of choice and presentation of alternatives); competence support (i.e., positive feedback related to a specific task); relatedness support (i.e., demonstration of emotional support); autonomy thwarting (i.e., use of controlled rewards); competence thwarting (i.e., expression of behaviors that emphasize guilt and doubt) and relatedness thwarting (i.e., perception of behaviors of rejection). When perceiving supportive interpersonal behaviors, people perform more self-determined actions. Conversely, when experiencing thwarting behaviors, individuals tend to manifest less self-determined actions (Rocchi et al., 2016).

Some recent studies (e.g., Chang et al., 2016; Rocchi and Pelletier, 2018) have shown that coaches who adopt supporting behaviors create conditions for individuals to self-regulate their own behavior, while decreasing less self-determined motivation. Moreover, autonomy support behaviors of the fitness instructors perceived by the participants have been positively associated with BPN satisfaction (Edmunds et al., 2007). Inversely, thwarted interpersonal behaviors adopted by exercise professionals may lead into BPN frustration or more controlled motivational regulations (Ng et al., 2013).

Researchers clearly affirm the relevance of deeper understanding of exercisers' perception on interpersonal behaviors (i.e., supportive and thwarting) of exercise professionals and how they might influence BPN satisfaction or frustration (Puente and Anshel, 2010; Bartholomew et al., 2011b). Some studies show that exercise professionals may influence individual's well-being, as well as persistence and adherence to practice (Vlachopoulos and Karavani, 2009). According to these authors, highly supportive profiles expressed by exercise professionals are important on individuals exercise maintenance over the long run.

As of today, merely one systematic review in the exercise context (Teixeira et al., 2012), having SDT as theoretical background, has analyzed the satisfaction (although not frustration) of basic psychological needs, the motivational regulation, and the way these can predict different physiological outcomes (i.e., energy expenditure, body mass index). Nevertheless, these authors did not study in greater detail exercisers' perception of exercise professionals' interpersonal behaviors. In addition, a search on Web of Science, using the keywords “self-determination” and “exercise,” revealed the existence of 650 new entries, published between the date of publication of the systematic review made by Teixeira et al. (2012) and this review. These findings not only sustain the privileged status acquired by the SDT regarding the understanding of the role of motivational variables on exerciser's behaviors, but also emphasizes the importance of explaining the links between different motivational variables, in order to figure out how interpersonal behaviors influence satisfaction or frustration of BPN's that, consequently, impact motivation quality and future behaviors outcomes.

Therefore, the purpose of this review is to analyze the associations between motivational variables (interpersonal behaviors, BPN satisfaction and frustration and motivational regulations) and behavioral outcomes (i.e., enjoyment, intention, persistence and adherence), in healthy exercisers having as conceptual background SDT.

## Methods

The several stages of the present review followed recommendations suggested by the PRISMA protocol (Moher et al., 2009).

### Research strategy

A broad search of literature was conducted on the following databases: Web of Science, PubMed, SportDISCUS and PsycINFO; from December 23, 2017 until April 30, 2018. Keywords that have been used are “interpersonal behavior,” “behavior^*^ regulation,” “basic needs,” “need satisfaction,” “need frustration,” “motiv^*^ regulation,” “motiv^*^,” “enjoyment,” “exercise^*^,” “ intention,” “persistence,” “adherence,” “health clubs,” “gym,” “fitness.” These have been used separately or in different combinations, through the inclusion of “AND” or “OR.” Bibliographic references were examined in an attempt to include potential studies that met inclusion criteria.

### Inclusion/exclusion criteria

The following inclusion criteria were adopted: (1) experimental and non-experimental studies; (2) published between August 1985 and April 2018 (date of first publication on SDT; date of the end of data collection); (3) written in English; (4) based on SDT; (5) including at least one of the studied variables (interpersonal behavior, basic psychological needs, motivational regulation, enjoyment, intention, persistence, and adherence); (6) sampling exercisers, aged between 18 and 65 years; (7) focusing on apparently healthy individuals (studies that included overweight and/or obese people were also included). Exclusion criteria were as follows: (1) studies published after May 2018; (2) including amateurs or professional athletes, since sport, and physical exercise are distinguished concepts (Caspersen et al., 1985); (3) published in physical education classes, since this type of physical activity is different from regular exercise (Caspersen et al., 1985); (4) instrument validation studies; (5) gray literature; (6) evaluation of physiological factors unrelated to previously mentioned variables; (7) systematic reviews.

### Data extraction

Data was extracted by one of the authors using a predefined checklist and was verified and analyzed by two other authors. The following information was extracted: (1) bibliographic information (authors, year of publication, country of research), (2) study design; (3) sample characteristics; (4) instruments; (5) motivational variables predictors; (6) main results; (7) statistical analysis.

### Qualitative analysis of the studies methods

Checklist created by Black and Downs (1998) was used to qualitatively evaluate studies' methodological content. This instrument consists of 27 questions that seek to determine the study's quality by having in mind several parameters, namely study design, adequacy of statistical procedures, description clarity of the main conclusions. However, since one question (question 15—*was an attempt made to blind measuring the main outcomes of the intervention?)* was not applicable to all studies analyzed, it was removed from the original checklist. Therefore, the modified scale had maximum 26 points from the original one (item 15 was excluded, maximum result: 26). Two reviewers analyzed the selected studies and any discrepancies were resolved consensually.

### Additional analysis

To facilitate the process of analyzing SDT predictors with behavioral outcomes, we used the system created by Teixeira et al. (2012), since it is very simple and practical. If 75% of the sample showed positive associations with behavioral outcomes (e.g., exercise frequency) they were codded with “++”, and “+” for percentages between 75% and 50%. Negative associations above 75% were codded “––”, and “–” for percentages between 75 and 50%. Null positive associations “0/+” or null negative associations “0/–” where coded if the evidence was divided between any association and positive or negative, respectively.

## Results

### Study selection

**Figure 1:**
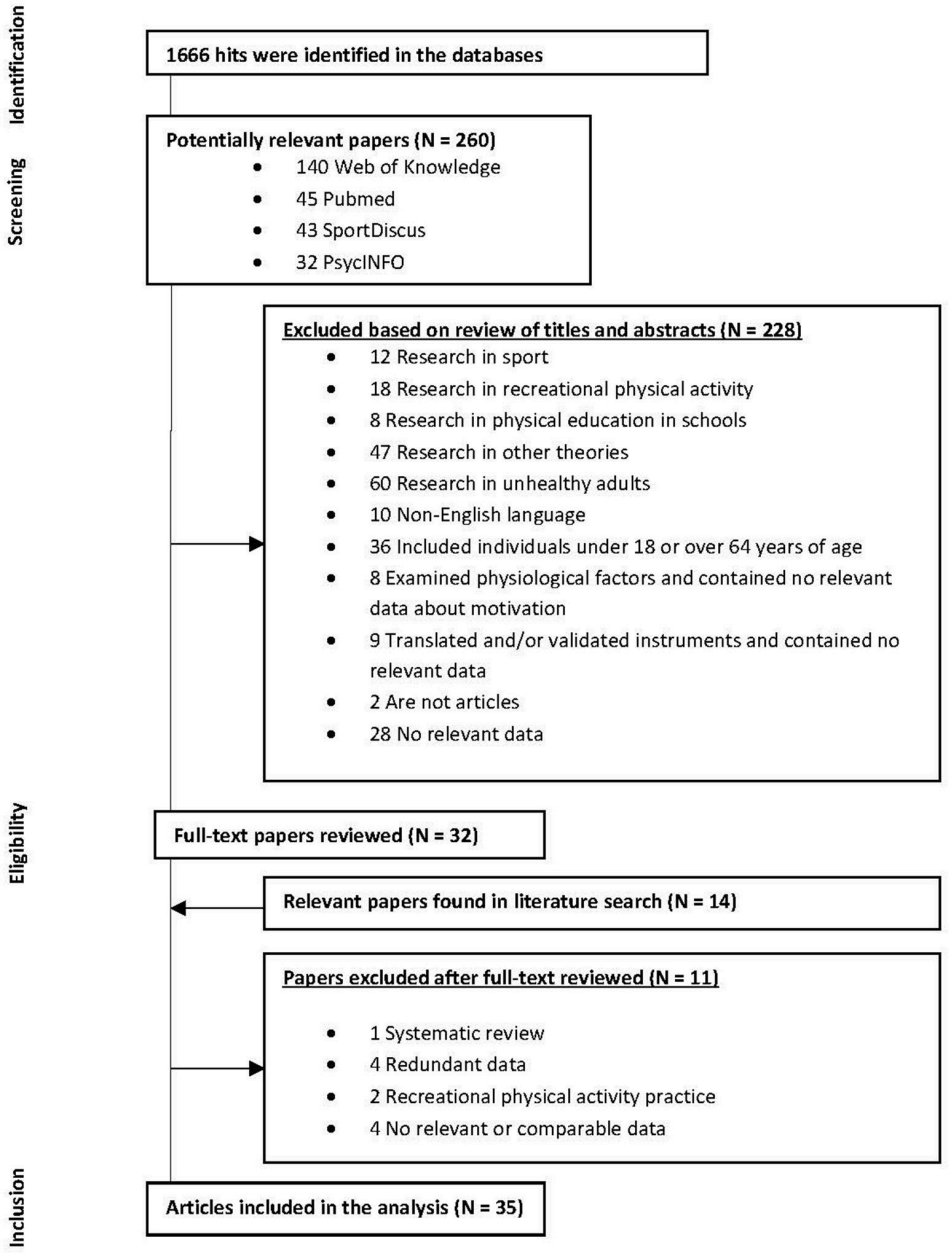
Studies chart flow.

During research (Figure 1), a total of 1,666 titles were identified, 260 of which were selected as they appeared to be potentially relevant for this systematic review. After carefully reading the titles and abstracts of the abovementioned articles, the selection was shortcut to 32 articles. By analyzing their bibliographical references, 14 other potentially relevant articles on the topic were pinpointed, leading to a total of 46 articles selected, which were fully and attentively analyzed. Studies that did not meet the previously stated inclusion criteria were excluded (*n* = 11). The final sample consisted, therefore, of 35 articles, of which 19 (54%) are cross-sectional, 12 (34%) experimental, 3 (9%) perspective, and 1 (3%) retrospective.

### Study summaries

The present review includes 35 empirical studies published until April 2018. All studies based on SDT interventions in the exercise domain were evaluated. Prospective, experimental, and cross-sectional studies that examined interpersonal behaviors, BPN satisfaction and frustration, motivation regulations, and their impact on behavioral outcomes (e.g., enjoyment, intention, persistence, adherence) were included. Table 1 presents a synthesis of the data extracted from the 35 studies comprehended in this review. Studies are listed by SDT construct (i.e., interpersonal behaviors, BPN satisfaction and frustration, motivational regulation) and alphabetically organized by author's name.

**Table 1:** Description of reviewed studies.

**Author (date)**	**Location**	**Design**	**Size (%F)**	**Features (M ± DP)**	**Measures**	**Significant predictors**	**Outcomes**	**Analysis**	**Quality**
**INTERPERSONAL BEHAVIOR**									
Edmunds et al., 2006	UK	Cross-sectional	369(52)	Exercisers (31.86 ± 11.28)	Perceived Autonomy Support (HCCQ)	MV: Autonomy support (+)	Psychological need satisfaction (autonomy, competence, relatedness)	Hierarchical multiple regression analysis	15
Edmunds et al., 2007	UK	Prospective (3 months)	49(84)	Overweight/Obese (44.98 ± 14.61)	Perceived Autonomy Support (HCCQ)	MV: Autonomy support (+)	Exercise self-regulations (identified and integrated regulation, intrinsic motivation)	Hierarchical regression analysis	18
Edmunds et al., 2008	UK	Experimental (10 weeks)	56(100)	Exercisers (31.86 ± 11.28)	Autonomy Support (PESS)	MV: CG Autonomy support (-), EG Autonomy support (+), over time	Intention	Multiple regression analysis	19
Klain et al., 2015	Brazil	Cross-sectional	588(65)	Exercisers (35.00 ± 17.00)	Perceived Autonomy Support (PASECQp)	MV: Autonomy support (+)	Psychological need satisfaction (autonomy, competence, relatedness), Adherence	MANOVA	17
Moreno-Murcia et al., 2016	Spain	Cross-sectional	355(100)	Exercisers (35.30 ± 12.20)	Perceived Autonomy Support (PASSES)	BIV: Autonomy support (+)	Psychological need satisfaction (autonomy, competence, relatedness), Self-reported exercise (total and strenuous PA)	Bivariate correlations; SEM	8
Ng et al., 2013	UK	Retrospective Gagn (6 months)	156(80)	Exercisers (31.01 ± 13.21)	Perceived Autonomy Support (HCCQ)	MV: Autonomy support (+)	Psychological need satisfaction (autonomy, competence, relatedness), Self-reported exercise (total and strenuous PA)	Bivariate correlations; One-way ANOVA	17
Ntoumanis et al., 2016	UK	Experimental (4 months)	364(68)	Instructors (37.28 ± 7.65) + Exercisers (39.88 ± 13.12)	Perceived Autonomy Support (HCCQ)	MV: Autonomy support (+)	Intention	MANOVA	25
Puente and Anshel, 2010	USA	Cross-sectional	238(57)	Healthy individuals (20.4 ± 2.16)	Perceived Autonomy Support (SCQ) + Competence Support (PCS)	MV: Autonomy support (+), Competence support	Psychological need satisfaction (autonomy, competence), Adherence	Bivariate correlations; SEM	6
Silva et al., 2011	Portugal	Experimental (1 + 2 year follow-up)	221(100)	Overweight/Obese (44.98 ± 14.61)	Perceived Autonomy Support (HCCQ)	BIV: Autonomy support (+)	Exercise self-regulations (autonomous motivation), Adherence	Effect sizes; Independent *T*-test; SEM	22
Silva et al., 2010	Portugal	Experimental (12 months)	239(100)	Overweight/Obese (intervention: 38.1 ± 7.04; control 37.1 ± 6.99)	Perceived Autonomy Support (HCCQ)	BIV: Autonomy support (+)	Psychological need satisfaction (autonomy, competence), Adherence	Effect sizes; Independent *T*-test; SEM	11
Vlachopoulos et al., 2011	Greece	Cross-sectional	733(40)	Exercisers (18–64year)	Perceived Autonomy Support (PAS)	MV: Autonomy support (n.s.)	Psychological need satisfaction (autonomy, competence, relatedness)	Hierarchical regression analysis	9
**BASIC PSYCHOLOGICAL NEED SATISFACTION AND FRUSTRATION**									
Edmunds et al., 2006	UK	Cross-sectional	369(52)	Exercisers (31.86 ± 11.28)	Psychological Need Satisfaction (BNSWS adapted)	BIV: Autonomy (+), Competence (+), Relatedness (+)	Self-reported exercise (total PA)	Bivariate correlations; Regression analysis	15
Edmunds et al., 2007	UK	Prospective (3 months)	49(84)	Overweight/Obese (44.98 ± 14.61)	Psychological Need Satisfaction (PNSS)	MV: Autonomy (n.s.), Competence (n.s.), Relatedness (n.s.)	Adherence	Multiple regression analysis; Paired *T*-test	18
Edmunds et al., 2008	UK	Experimental (10 weeks)	56(100)	Exercisers (31.86 ± 11.28)	Psychological Need Satisfaction (PNSS)	MV: Autonomy (+), Competence (+), Relatedness (+)	Adherence	Multiple regression analysis	19
Klain et al., 2015	Brazil	Cross-sectional	588(65)	Exercisers (35.00 ± 17.00)	Psychological Need Satisfaction (BPNESp)	MV: Autonomy (+), Competence (+), Relatedness (n.s.)	Adherence	MANOVA	17
Moreno-Murcia et al., 2016	Spain	Cross-sectional	355(100)	Exercisers (35.30 ± 12.20)	Psychological Need Satisfaction (BNSE)	MV: BPN (+)	Exercise self-regulations (intrinsic motivation); Self-reported exercise (total and strenuous PA)	Bivariate correlations; SEM	8
Ng et al., 2013	UK	Retrospective (6 months)	156(80)	Exercisers (31.01 ± 13.21)	Psychological Need Satisfaction (BNSE) + Frustration (PNTS)	MV: BPN satisfaction (n.s.); BPN frustration (n.s.)	Adherence	Bivariate correlations; One-way ANOVA	17
Ntoumanis et al., 2016	UK	Experimental (4 months)	364(68)	Instructors (37.28 ± 7.65) + Exercisers (39.88 ± 13.12)	Psychological Need Satisfaction (BPNES)	MV: Autonomy (+), Competence (n.s.), Relatedness (+)	Intention	MANOVA	25
Puente and Anshel, 2010	USA	Cross-sectional	238(57)	Healthy individuals (20.4 ± 2.16)	Psychological Need Satisfaction (BPNS)	MV: Autonomy (+), Competence (+), Relatedness not measured	RAI, Adherence	Bivariate correlations; SEM	6
Puigarnau et al., 2017	Spain	Experimental (6 months)	56(87)	Healthy individuals (36.02 ± 10.15)	Psychological Need Satisfaction (PNSE)	MV: EG: Autonomy (n.s.), Competence (n.s.), Relatedness (n.s.), CG: Autonomy (+), Competence (n.s.), Relatedness (n.s.)	Adherence	Bivariate correlations; independent *T*-test; MGM	7
Silva et al., 2010	Portugal	Experimental (12 months)	239(100)	Overweight/Obese (intervention: 38.1 ± 7.04; control 37.1 ± 6.99)	Psychological need satisfaction (LCE + IMI)	MV: Autonomy (+), Competence (+), Relatedness not measured	Exercise self-regulations (introjected, identified and intrinsic motivation)	Effect sizes; Independent *T*-test; SEM	11
Sylvester et al., 2018	Canada	Cross-sectional	499(65)	Healthy individuals (female: 34.09 ± 13.27; male: 33.43 ± 12.84)	Psychological Need Satisfaction (PNSE)	MV: PNS (+)	Exercise self-regulations (autonomous motivation)	Multiple mediation analysis; SEM	7
Teixeira et al., 2018	Portugal	Cross-sectional	153(55)	Exercisers (36.21 ± 8.44)	Psychological Need Satisfaction (PNSE) + Frustration (PBNSFS adapted)	MV: PNS (+), PNF (–)	Exercise self-regulations (identified, integrated and intrinsic motivation)	Multiple mediation analysis; SEM	11
Teixeira and Palmeira, 2015	Portugal	Cross-sectional	905(61)	Exercisers (36.8 ± 11.96)	Psychological Need Satisfaction (PNSE)	MV: PNS (+)	Emotional outcome (psychological well-being)	Multiple mediation analysis; SEM	15
Vlachopoulos et al., 2011	Greece	Cross-sectional	733(40)	Exercisers (18–64year)	Psychological Need Satisfaction (PNES)	MV: Autonomy (+), Competence (+), Relatedness (+)	Exercise self-regulations (introjected, identified and intrinsic motivation)	Hierarchical regression analysis	9
Vlachopoulos and Neikou, 2007	Greece	Prospective (6 months)	228(52)	Exercisers (F: 27.79 ± 6.28; M: 27.38 ± 8.37)	Psychological Need Satisfaction (BPNES)	MV: F: Autonomy (+), Competence (+), Relatedness (n.s.), M: Autonomy (n.s.), Competence (+), Relatedness (n.s.)	Adherence	Hierarchical regression analysis; SEM	9
**MOTIVATIONAL REGULATION**									
Blais et al., 2017	Canada	Experimental (6 months)	70(76)	Exercisers (44.83 ± 8.78)	Exercise self-regulations (BREQ-3)	MIV: IM (+), INTE (+), IDEN (n.s.), INTR (+), EXT (–), AMO not measured	Change in exercise stages	Mixed Model ANOVA	9
Caudwell and Keatley, 2016	Australia	Cross-sectional	100(0)	Exercisers (25.83 ± 6.62)	Perceived Locus of Causality (PLOC)	BIV: AUTO (+), CON (n.s.)	Attendance (adherence)	Hierarchical regression analysis	15
Duncan et al., 2010	Canada	Cross-sectional	1,079(57)	Exercisers (24.15 ± 9.61)	Exercise self-regulations (BREQ-3)	BIV: IM (n.s.), INTE (+), IDEN (+), INTR (n.s.), EXT (n.s.), AMO (n.s.)	Frequency (adherence)	Multiple regression analysis	11
Edmunds et al., 2006	UK	Cross-sectional	369(52)	Exercisers (31.86 ± 11.28)	Exercise self-regulations (BREQ)	BIV: IM (+), IDEN (+), INTR (+), EXT (–)	Self-reported exercise (total PA)	Hierarchical regression analysis; Mediation analysis	15
Edmunds et al., 2007	UK	Prospective (3 months)	49(84)	Overweight/Obese (44.98 ± 14.61)	Exercise self-regulations (BREQ-2 + EMS)	BIV: IM (+), INTE (+), IDEN (–), INTR (+), EXT (–), AMO not measured	Self-reported exercise (total PA), intention, persistence	Bivariate correlations; Multiple regression analysis	18
Edmunds et al., 2008	UK	Experimental (10 weeks)	56(100)	Exercisers (31.86 ± 11.28)	Exercise self-regulations (BREQ-2 + EMS)	MIV: IM (+), INTE (+), IDEN (n.s.), INTR (+), EXT (n.s.), AMO (–)	Intention, adherence, CG: decreased over time; EG: increased over time	Multilevel regression analysis	19
Gast et al., 2011	USA	Cross-sectional	181(0)	Healthy individuals (21.43 ± 3.66)	Exercise self-regulations (BREQ)	BIV: IM (+), IDEN (+), INTR (n.s.), EXT (n.s.)	Self-reported exercise (total and strenuous PA), Eating behavior	Independent *T*-test; Linear regression analysis	14
Heiestad et al., 2016	Norway	Experimental (12 weeks)	143(100)	Overweight/Obese (39.90 ± 10.50)	Exercise self-regulations (BREQ-2)	BIV: RAI (+), IDEN (+), INTR (+), EXT (n.s.), AMO (n.s.), IM not measured	Self-reported exercise (quality of life), Adherence	One-way ANOVA	26
Ingledew and Markland, 2008	UK	Cross-sectional	252(48)	Healthy individuals (40.36 ± 10.96)	Exercise self-regulations (BREQ-2)	BIV: IM (n.s.), IDEN (+), INTR (n.s.), EXT (–), AMO not measured	Self-reported exercise (total and strenuous PA)	Bivariate correlations; SEM	11
Ingledew et al., 2009	UK	Cross-sectional	251(52)	University students (19.48 ± 1.90)	Exercise self-regulations (BREQ-2)	MIV: IM (+), IDEN (+), INTR (n.s.), EXT (n.s.), AMO not measured	Self-reported exercise (total and strenuous PA)	Multiple regressions analysis; SEM	10
Klain et al., 2015	Brazil	Cross-sectional	588(65)	Exercisers (35.00 ± 17.00)	Exercise self-regulations (BREQ-2)	BIV: IM (+), IDEN (+), INTR (n.s.), EXT (–), AMO (-)	Adherence	MANOVA; SEM	17
Mack et al., 2015	Canada	Cross-sectional	465(49)	Healthy individuals (20.55 ± 1.75)	Exercise self-regulations (BREQ-3)	MIV: IM (+), INTE (+), IDEN (+), INTR (+), EXT (–), AMO not measured	Self-reported exercise (total and strenuous PA)	Multiple mediation analysis	12
Mullan and Markland, 1997	UK	Cross-sectional	314(50)	Healthy individuals (female: 36.04 ± 11.07; male: 39.07 ± 11.45)	Exercise self-regulations (BREQ)	BIV: IM (+), IDEN (+), INTR (n.s.), EXT (–)	Change in exercise stages	Bivariate correlations analysis	7
Ntoumanis et al., 2017	Greece	Experimental (14 weeks)	180(56)	Exercisers (30.10 ± 9.60)	Exercise self-regulations (BREQ-2)	MIV: IM (+), IDEN (+), INTR (+), EXT (n.s.), AMO (–), increased over time	Exercise identity	MGM	10
Ntoumanis et al., 2016	UK	Experimental (4 months)	364(68)	Instructors (37.28 ± 7.65) + Exercisers (39.88 ± 13.12)	Exercise self-regulations (BREQ)	BIV: AUTO (n.s.), CON (n.s.), AMO (n.s.)	Intention	MANOVA	25
Puente and Anshel, 2010	USA	Cross-sectional	238(57)	Healthy individuals (20.4 ± 2.16)	Exercise self-regulations (SRQ-E)	MV: RAI (+)	Adherence, enjoyment	Bivariate correlations; SEM	6
Puigarnau et al., 2017	Spain	Experimental (6 months)	56(87)	Healthy individuals (36.02 ± 10.15)	Exercise self-regulations (BREQ-3)	BIV: IM (n.s.), INTE (n.s.), IDEN (n.s.), INTR (n.s.), EXT (n.s.), AMO (n.s.), for both groups	Frequency (adherence)	Bivariate correlations; independent *T*-test	7
Rodgers et al., 2010	Canada	Prospective	1,572(60)	Healthy individuals (initiates vs. long-term exercisers, 22–51 year)	Exercise self-regulations (BREQ)	MV: IM (+), IDEN (+), INTR (n.s.), EXT (–), overtime for initiates, but < to long-term exercisers	Self-reported exercise initiate vs. long-term exercisers	MANOVA	8
Rosa et al., 2015	Brazil	Experimental (12 months)	73(78)	Exercisers (34.01 ± 9.71)	Exercise self-regulations (BREQ-2)	BIV: RAI (+), IM (+), IDEN (n.s.), INTR (–), EXT (n.s.), AMO (n.s.)	Adherence	Two-way ANCOVA	11
Sibley and Bergman, 2016	USA	Experimental (8 weeks)	461(60)	Healthy individuals (20.20 ± 2.30)	Exercise self-regulations (BREQ)	MIV: RAI (+), IM (+), IDEN (+), INTR (–), EXT (–)	Self-reported exercise (total PA)	Multiple regressions analysis; SEM	11
Silva et al., 2011	Portugal	Experimental (1 + 2 year follow-up)	221(100)	Overweight/Obese (44.98 ± 14.61)	Exercise self-regulations (SRQ-E)	BIV: IM (+), IDEN (+), INTR (n.s.), EXT (n.s.), higher 2-year follow-up	Adherence	Effect sizes; Independent *T*-test; SEM	22
Silva et al., 2010	Portugal	Experimental (12 months)	239 (100)	Overweight/Obese (intervention: 38.1 ± 7.04; control 37.1 ± 6.99)	Exercise self-regulations (SRQ-E)	BIV: IM (+), INTE (n.s.), IDEN (+), INTR (+), EXT (n.s.)	Adherence	Effect sizes; Independent *T*-test	11
Standage et al., 2008	UK	Cross-sectional	52(50)	Healthy individuals (22.27 ± 3.41)	Exercise self-regulations (BREQ)	BIV: IM (+), IDEN (+), INTR (n.s.), EXT (–)	Self-reported exercise (Total and strenuous PA)	Bivariate correlations	7
Sylvester et al., 2018	Canada	Cross-sectional	499(65)	Healthy individuals (female: 34.09 ± 13.27; male: 33.43 ± 12.84)	Exercise self-regulations (BREQ-3)	MIV: AUTO (+), CON not measured	Self-reported exercise (Total and strenuous PA)	Multiple mediation analysis; SEM	7
Teixeira et al., 2018	Portugal	Cross-sectional	153(55)	Exercisers (36.21 ± 8.44)	Exercise self-regulations (BREQ-3P)	MV: IM (+), INTE (+), IDEN (+), INTR (n.s.), EXT (–)	Emotional response to exercise	Multiple mediation analysis; SEM	11
Teixeira and Palmeira, 2015	Portugal	Cross-sectional	905(61)	Exercisers (36.8 ± 11.96)	Exercise self-regulations (BREQ-2)	MV: IM (+), IDEN (+), INTR (+), EXT (-), AMO (–)	Emotional outcome (psychological well-being)	Multiple mediation analysis; SEM	15
Thogersen-Ntoumani and Ntoumanis, 2006	UK	Cross-sectional	375(51)	Exercisers (38.7 ± 10.9)	Exercise self-regulations (BREQ + AMS)	MV: IM (+), IDEN (+), INTR (n.s.), EXT (–), AMO (–)	Intention, adherence	Multivariate regressions analysis; MANOVA	9
Thogersen-Ntoumani et al., 2015	Australia	Experimental	87(65)	Exercisers (42.00 ± 12.00)	Exercise self-regulations (BREQ-2)	MV: AUTO (+), CON (–), AMO (n.s.)	Body composition; Adherence	Repeated-measures ANOVA; SEM	13
Vlachopoulos et al., 2011	Greece	Cross-sectional	733(40)	Exercisers (18–64year)	Exercise self-regulations (BREQ-2)	MV: IM (+), IDEN (+), INTR (+), EXT (–), AMO (-)	Exercise identity	Confirmatory factor analysis	9
Wilson and Rodgers, 2004	Canada	Cross-sectional	276(64)	Healthy individuals (20.86 ± 2.21)	Exercise self-regulations (BREQ-2)	MV: IM (+), IDEN (+), INTR (+), EXT (–), AMO (–)	Intention	Multiple regressions analysis; SEM	11
Wilson et al., 2003	Canada	Experimental (12 weeks)	53(83)	Exercise practitioners (41.80 ± 10.75)	Exercise self-regulations (BREQ)	MV: IM (+), IDEN (+), INTR (n.s.), EXT (n.s.)	Self-reported exercise (total and strenuous PA)	Multiple regressions analysis	11

*F, female; M, Male; PA, Physical Activity; BIV, uni/bivariate associations; MV, multivariate associations; IM; intrinsic motivation; INTE, integrated regulation, IDEN, identified regulation; INTR, introjected regulation; EXT, external regulation; AMOT, amotivation; Auto, autonomous motivation; Cont, controlled motivation; PLOCaut, perceived locus of causality autonomous; PLOCcon, perceived locus of causality controlled; RAI, relative autonomy index; PNS, psychological need satisfaction; PNF, Psychological Need Frustration; n.s., not significant; +, positive relation; –, negative relation*.

### Characteristics of the studies

Table 1 summarizes descriptive data of the 35 articles analyzed. The vast majority of the samples consisted of regular exercisers and encompassed an extended age range (i.e., ages 18–64). The 35 studies included in this body of work englobed a total of 40 independent samples. The higher number of samples in comparison to the number of analyzed articles is explained by the fact that some studies have analyzed more than one sample (i.e., Rodgers et al., 2010). Altogether, the sample of this review consists of 10,482 healthy exercisers, predominantly female.

### Quality of the studies

Methodological quality of the studies was considered reasonable. Of the 26 existent criteria, the study with the highest matching number of criteria (25) was written by Heiestad et al. (2016). On the other hand, the article with the lowest number of fulfilled criteria (6) was published by Puente and Anshel (2010). For more details see Table 1.

### Additional analysis

Table 2 presents a summary of the sample characteristics (i.e., sample size, age, gender) of all 35 studies included in this review. In Table 3, we can observe the analysis made according to the classification system used in another study.

**Table 2:** Summary of samples characteristics.

**Characteristics**	**Sample *K* (%)**
**SAMPLE SIZE**	
≤100	12(30)
100–300	15(38)
300–500	9(22)
≥500	4(10)
**GENDER**	
Women only	5(13)
Men only	2(5)
Men and Women combined	33(82)
**LOCATION**	
America	18(45)
Europe	20(50)
Australia	2(5)
**MEAN AGE,YEARS**	
<25	12(30)
26–45	25(63)
46–65	2(5)
Unable to determine	1(2)
**FEATURES**	
Exercise practicioners	24(60)
Healthy individuals	7(17)
University students	5(13)
Overweight and obese	4(10)
**EXERCISE AND RELATED OUTCOMES**	
Self-reported exercise	13(26)
Change in exercise stages	1(2)
Intention	4(8)
Persistence	3(6)
Adherence	24(50)
Other^*^	4(8)
Total *K*	40

* *Exercise identity, eating behavior, enjoyment, emotional outcomes*.

**Table 3:** Summary of associations between SDT predictors and exercise outcomes.

	**Supporting associations**				
	***N***	**+ (%)**	**– (%)**	**0 (%)**	**Association**
**INTERPERSONAL BEHAVIOR**					
Autonomy support	11	11 (100)	0(0)	0(0)	++
**BASIC PSYCHOLOGICAL NEED SATISFACTION AND FRUSTRATION**					
Autonomy satisfaction	11	10(90)	0(00)	1(10)	++
Competence satisfaction	11	8(73)	0(0)	3(27)	+
Relatedness satisfaction	9	5(56)	0(0)	4(44)	+
Autonomy frustration	0*	0(0)	0(0)	0(0)	?
Competence frustration	0*	0(0)	0(0)	0(0)	?
Relatedness frustration	0*	0(0)	0(0)	0(0)	?
Composite satisfaction score	3	3(100)	0(0)	0(0)	++
Composite frustration score	2*	0(0)	1(50)	1(50)	?
**EXERCISE REGULATIONS/MOTIVATIONS**					
Intrinsic motivation	27	24 (89)	0(0)	3(11)	++
Integrated motivation	8	6 (75)	0(0)	2(25)	++
Identified regulation	27	21(78)	1(4)	5(18)	++
Introjected regulation	27	11(40)	2(8)	14(54)	0/+
External regulation	27	0 (0)	15(56)	12(44)	–
Amotivation	14	0(0)	6(43)	8(57)	–
Relative autonomy (e.g., RAI)	4	4(100)	0(0)	0(0)	++
Composite autonomous regulations score	4	3(75)	0(0)	1(25)	++
Composite controlled regulations score	2*	0(0)	1(50)	1(50)	?

N, number of studies; (++), positive associations for percentage ≥75% and (+) for percentages between 50 and 75%; (--) negative associations for percentage ≥75% and (–) for percentage between 50 and 75%; (0/+) null positive or (0/–) null negative associations when the evidence was split between no association (0) and either positive or negative associations, respectively; (?) for other results indicating inconsistent findings; *studies available <3.

A total of 11 studies analyzing practitioners' perception of exercise professionals' interpersonal behaviors, were included in this review. Of these studies, 5 are transversal, 4 experimental, 1 retrospective and 1 perspective. This review comprised 14 studies that analyzed the impact of BPN satisfaction and/or frustration on motivational regulations and/or behavioral outcomes. Thus, the vast majority (*n* = 32) of the studies integrated in this review addressed motivational regulations and their relationship with exercise behaviors (i.e., frequency, self-reported physical activity).

## Discussion

This review aimed to analyze the literature focused on the relationship between motivational variables and behavioral outcomes in the exercise context, having as a theoretical background Self-Determination theory. As postulated by this motivational construct, BPN's satisfaction and/or frustration is influenced by the individuals' surrounding environment. Moreover, this same environment also plays an important role when it comes to predict more or less self-determined regulations and influence the way a person manifests his/her behavior (Ryan and Deci, 2017).

By looking at the various articles included in this study, one might conclude that research in the exercise context, having as a theoretical background SDT, seems to have been exponentially growing in the recent years. As a matter of fact, 18 (51%) of the analyzed articles were written in the last 6 years (>2012), while the remaining ones were written previously (1997–2011), thus, demonstrating the increased interest in applying SDT in exercise context. That being written, this systematic review proposes an updated summary of investigations on this topic that has been done up until now, aiming to complement and enhance the previous existent review (Teixeira et al., 2012). In what follows, an analysis of the selected studies grouped by motivational variables, namely: interpersonal behaviors, basic psychological needs, and motivational regulation, is provided.

### Interpersonal behavior

According to this body of work, exercisers who perceive

autonomy and competence support from exercise professionals tend to have greater BPN satisfaction (Edmunds et al., 2006; Puente and Anshel, 2010; Silva et al., 2010; Vlachopoulos et al., 2011; Ng et al., 2013; Klain et al., 2015; Moreno-Murcia et al., 2016). These results are in line with the Self-determination theory's theoretical assumptions, which suggest that support for autonomy, competence and relatedness may be predictors of BPN satisfaction (Deci and Ryan, 2000). Therefore, it seems fundamental that exercise professionals are able to create a supportive context for BPN satisfaction, hence avoiding behavior dropout.

Research demonstrated that there are positive relationships between autonomy support and more autonomous regulations (Edmunds et al., 2008; Silva et al., 2011). In addition, significant relationships between autonomy support and intention to exercise appears to exist (Edmunds et al., 2008; Ntoumanis et al., 2016). This is equally true regarding adherence, which is positively associated with the support of autonomy perceived by overweight and obese exercisers (Silva et al., 2010, 2011). Overweight and obese exercisers who perceived autonomy support from their exercise professionals, show more autonomous forms of motivation (Edmunds et al., 2007). As a matter of fact, exercisers who feel support from their exercise professionals in decision making tend to maintain exercise practice in the long term. In addition, several studies (Ng et al., 2013; e.g., Moreno-Murcia et al., 2016) show that individuals who perceive greater autonomy support tend to practice more exercise with higher intensities.

However, no study has considered the six dimensions of interpersonal behavior that can be perceived by exercisers, regarding exercise professionals' behaviors. This may be partly related to the lack of validated instruments that englobe all three dimensions of support and of thwarting. According to several authors (Ryan and Deci, 2017; e.g., Rocchi et al., 2017), the analysis of all six interpersonal behaviors' constructs and the way each dimension influences BPN satisfaction or frustration proves to be essential. Only recently, Rocchi et al. (2017) developed and validated for the first time the Interpersonal Behavior Questionnaire (IBQ) and the Interpersonal Behavior Questionnaire - Self (IBQ-Self), a scale aiming at analyzing, respectively, students' perception of teachers' behaviors and the perception teachers have about their own behaviors. However, this scale has not yet been used or validated in the exercise context, which may be related, in part, to the lack of research analyzing the dimensions of support and thwarting of interpersonal behaviors.

### Basic psychological needs

The studies provided good evidence on the positive correlation among BPN satisfaction, a more autonomous and self-determined motivation and behavior maintenance. The findings of Edmunds et al. (2008) assumed a paramount relevance by evidencing the predictive role of BPN satisfaction in facilitating the process of self-determination. These authors concluded that exercisers, who experience psychological need's fulfillment, tend to demonstrate a greater autonomous motivation. It is also true in overweight and obese exercisers, where BPN's satisfaction was related to more self-determined motivational regulations (Silva et al., 2010). In point of fact, this proves to be consistent with SDT, which emphasizes that autonomous regulations are fostered by the satisfaction of the basic psychological needs (Deci and Ryan, 2000).

Moreover, when experiencing satisfaction of BPN's, exercisers tend to manifest greater adherence (Edmunds et al., 2007, 2008; Puente and Anshel, 2010; Ng et al., 2013; Klain et al., 2015; Teixeira and Palmeira, 2015) and greater frequency of self-reported exercise (Edmunds et al., 2006). When examining each BPN separately, findings notice that autonomy satisfaction is a strong predictor of exercise intention (Edmunds et al., 2008; Teixeira et al., 2012). Therefore, individuals who perceive freedom of choice are more prone to maintain a long-term exercise practice. In addition, competence satisfaction is positively related with adherence (Puente and Anshel, 2010). Thus, individuals who acquire new skills or improve existing ones tend to have greater predisposition to maintain exercise frequency (Klain et al., 2015). Finally, relatedness satisfaction presents the lowest number of positive associations, which may be in part related to the preference of some exercisers to train alone (Klain et al., 2015; Moreno-Murcia et al., 2016; Puigarnau et al., 2017).

Only two of the analyzed articles focused on the impact of BPN frustration on motivational and emotional variables (Ng et al., 2013; Teixeira et al., 2018) and only one of them explored their impact on adherence to exercise (Ng et al., 2013). According to this studies, BPN frustration is a predictor of exercise dropout. These data corroborate other investigations, namely in sport (Sarrazin et al., 2002) or physical activity (Chatzisarantis and Hagger, 2009). However, these studies used different instruments that have not yet been validated in the exercise context. In addition, the small amount of studies focused on analyzing BPN frustration reveals the need for cautiously interpretations.

The frustration of basic psychological needs should be considered as an important variable to be measured (Vansteenkiste and Ryan, 2013) in order to fully understand its impact on motivational regulation and behavioral outcomes in exercise context. Not only to control BPN satisfaction, but to understand the possible existence between needs' frustration, less self-determined regulation, and exercise dropout.

Thus, by taking into consideration the analysis of BPN satisfaction and frustration, these studies are door openers for future research and highlighters of the need to create a specific instrument of analysis for the exercise context.

### Motivational regulation

As mentioned earlier, the majority of the studies addressed motivational regulations. In addition, they analyzed the impact of these motivational regulations on behavior maintenance, persistence, and adherence. A large percentage (84%) of them focused on all forms of motivational regulation, while others used composite factors of autonomous and controlled regulations (Thogersen-Ntoumani et al., 2015) or adopted the Relative Autonomy Index (Sibley and Bergman, 2016).

The results demonstrate a significant relationship between more autonomous regulations and exercise practice (Wilson et al., 2003; Edmunds et al., 2006, 2007; Ingledew and Markland, 2008; Standage et al., 2008; Ingledew et al., 2009; Rodgers et al., 2010; Mack et al., 2015; Heiestad et al., 2016; Sibley and Bergman, 2016; Blais et al., 2017; Sylvester et al., 2018), enhanced well-being (Teixeira and Palmeira, 2015), enhanced intention to exercise practice (Wilson and Rodgers, 2004; Thogersen-Ntoumani and Ntoumanis, 2006; Edmunds et al., 2008; Ng et al., 2013) and greater adherence (Puente and Anshel, 2010; Silva et al., 2010, 2011; Rosa et al., 2015). Therefore, exercisers, whose motivation is more self-determined, tend to maintain their behavior, hence being more prone to exercise over the long-run. Consequently, by engaging in an autonomous and volitional behavior, the exerciser might experience positive outcomes such as the feeling of enjoyment (Puente and Anshel, 2010), greater physical capacity (Sibley et al., 2013), enhanced body transformation (Thogersen-Ntoumani et al., 2015), and increased exercising frequency (Duncan et al., 2010; Caudwell and Keatley, 2016).

Intrinsic motivation represents the most self-determined regulation (Deci and Ryan, 2000). According to Table 3, this type of regulation mainly shows positive associations favoring different exercise behaviors, with solely three studies presenting no significant results. As a matter of fact, intrinsic motivation plays a major role for the exercisers to be able perform exercise spontaneously, to experience pleasure, to challenge themselves and to facilitate the behavior maintenance in the long term (Edmunds et al., 2008; Gast et al., 2011; Blais et al., 2017).

Integrated regulation prevails the least studied dimension, which might be justified by the fact that most of them so far cannot yet distinguish this regulation from identified regulation, given that both share the same principles (Teixeira et al., 2012). Integrated regulation was firstly analyzed in the review made by Wilson et al. (2006) on the Behavioral Regulation Exercise Questionnaire 2 (BREQ2: Markland and Tobin, 2004). Although 23 new studies have been published after the review from Wilson et al. (2006), nearly all of them used the previous version of BREQ-2, which does not englobe integrated regulation. Nevertheless, clear findings suggest a robust relationship between this type of motivational regulation, exercise intention (Edmunds et al., 2007, 2008) and exercise frequency (Duncan et al., 2010).

Besides, some studies (Edmunds et al., 2006; Thogersen-Ntoumani and Ntoumanis, 2006; Ingledew and Markland, 2008) advocate that identified regulation may be one of the strongest correlations in exercise context, which may be related in part to the effort required for exercise practice. In point of fact, identified regulation has been a key variable in predicting the maintenance of exercise behavior (Teixeira et al., 2012). This may be in part related to the positive health benefits that the individual perceives by exercising.

With regard to controlled regulation, studies show inconsistent results, therefore toughening the analysis. Some studies show significant differences between controlled regulation and behavioral outcomes (Edmunds et al., 2007), in others none (Duncan et al., 2010), and in some negative associations were found (Rosa et al., 2015). However, the literature suggests that this form of regulation is usually associated with negative adaptations such as feelings of guilt and pressure (Ryan and Deci, 2000a). People who perceive pressure to engage in exercise are more likely to feel guilty or ashamed if they do not exercise, thus, jeopardizing the potential of experiencing feelings of pleasure and enjoyment (Edmunds et al., 2006; Teixeira et al., 2012).

The positive results in the relationship between introjected regulation and exercise may be associated with an initial phase of self-determination (Gast et al., 2011; Ntoumanis et al., 2016), during the one the perception of the behavior is altered thanks to the recognition of the benefits associated with it, culminating in a greater potential for exercise habit implementation.

External regulation, the least self-determined one, is associated with behavioral dropout (Klain et al., 2015; Blais et al., 2017). Thus, when individuals engage in exercise practice only as a mean of obtaining an external reward, the chances of dropping out the behavior increase dramatically, as the results become dependent of factors one cannot control (Rodgers et al., 2010). Hence, in order to promote the behavior maintenance, the exerciser must recognize the physiological, psychological and emotional benefits of exercising. Only then, can the behavior maintenance be guaranteed (Edmunds et al., 2008).

Finally, amotivation, lying at one end of the motivational continuum, was one of the less studied regulation. This type of regulation was first analyzed in BREQ-2 (Markland and Tobin, 2004), after researchers realized that individuals may demonstrate unwillingness to exercise or that the reasons for their commitment have become less clear. According to this review, data shows a negative association between amotivation, persistence and adherence to exercise (Thogersen-Ntoumani and Ntoumanis, 2006; Thogersen-Ntoumani et al., 2015), which seems somehow expected, since lack of motivation is defined by the absence of the performance of a certain behavior.

### Limitations

During the analysis of the studies, some limitations that might influence data interpretation were observed. One of these constraints is related with the lack of a valid instrument that analyzes all six dimensions of interpersonal behavior in the exercise context. The employment of instruments created for other contexts compromises the comparison between studies, thus, stressing out the necessity to create and validate scales that can serve as universal method in comparing interpersonal behaviors' perceptions and the way they affect persistence and exercise practice. Future research may also work on the understanding of the relationship between supporting interpersonal behaviors and BPN satisfaction, or the interpersonal behaviors thwarting and BPN frustration. Additionally, we must bear in mind reduced amount of data related with BPN frustration and its impact on motivational regulations (Ng et al., 2013; Teixeira et al., 2018). As analyzed in this review, these studies addressed the composite values of BPN frustration, using instruments yet to be validated in exercise context. As previously stated, the use of instruments developed in other contexts, without prior validation for the domain being studied, might result in skewed results, and lead to biased interpretations of the data. Therefore, the validation of an instrument that analyzes BPN frustration urges. Only then, will a closer look at the relationship between frustration and motivational regulation, as well as on the way the person behaves before exercise, be possible. Furthermore, although more self-determined regulations predict intention to exercise, greater persistence, and adherence, there is still a need to examine this relationship in greater depth through longitudinal studies. In addition, studies focusing on emotional outcomes (i.e., enjoyment) derived from more autonomous regulations in exercise seem to be essential, given the lack of a significant number of investigations analyzing this behavior outcome.

We also suggest future research on analyzing in more detail (e.g., systematic reviews, meta-analysis) clinical trials developed with this type of population or in individuals with chronic diseases. This kind of investigation will increase our knowledge about the influence of exercise on behavioral outcomes, and how these exercise habits can improve health markers, having as theoretical background SDT.

Finally, despite the existence of numerous cross-sectional studies that demonstrate positive relationship between motivational variables and behavioral consequences in the physical exercise domain, longitudinal research that analyzes interpersonal behaviors and their relationship with persistence and adherence in exercisers appears to be of the utmost importance.

## Conclusion

This review presents the most current evidence for understanding SDT in exercise context and how this motivational construct appears to promote persistence and adherence to long-term practice. Overall, there is good evidence of the positive influence that autonomy support perceived by exercisers has in the satisfaction of the basic psychological needs. Similarly, the development of more autonomous regulations also appears to be linked to autonomy support behaviors. In addition, results analyzed in this study show that autonomous regulations predict greater intentions for exercise, regardless of age, group and nationality of the participants being englobed in the sample. To sum up, SDT affirms that supporting interpersonal behaviors perceived by individuals can strongly influence long-term exercise adherence.

## Author contributions

FR conceived this manuscript and led the writing team. FR and DM conducted the study search, summarized the quantitative review, and drafted the Results section. TB and LC made substantial contributions to the Discussion section. HPN, DT, JM, and DAM revised the entire manuscript and made important contributions in various sections. All authors read and approved the final version of the manuscript.

### Conflict of interest statement

The authors declare that the research was conducted in the absence of any commercial or financial relationships that could be construed as a potential conflict of interest.
